# Cytotoxic and antimicrobial effects of biosynthesized ZnO nanoparticles using of *Chelidonium majus* extract

**DOI:** 10.1007/s10544-017-0233-9

**Published:** 2017-11-27

**Authors:** Renata Dobrucka, Jolanta Dlugaszewska, Mariusz Kaczmarek

**Affiliations:** 1 Department of Industrial Products Quality and Ecology, Faculty of Commodity Science, Poznan University of Economics, al. Niepodległości 10, 61-875 Poznan, Poland; 2 Department of Genetics and Pharmaceutical Microbiology, Poznan University of Medical Sciences, 4 Swiecickiego Street, 60-781 Poznan, Poland; 3 Department of Immunology, Chair of Clinical Immunology, Poznan University of Medical Sciences, Rokietnicka 5D, 60-806 Poznan, Poland

**Keywords:** Zno nanoparticles, Antimicrobial activity, Cytotoxicity

## Abstract

The basic goal of this study was to synthesize zinc oxide nanoparticles using the *Chelidonium majus* extract and asses their cytotoxic and antimicrobial properties. The synthesized ZnO NPs were characterized by UV-Vis, Scanning Electron Microscopy (SEM) with EDS profile, Fourier Transform Infrared Spectroscopy (FTIR), X-ray diffraction (XRD), Transmission Electron Microscopy (TEM) and Atomic Force Microscopy (AFM). The aforementioned methods confirmed that the size of synthesized ZnO nanoparticles was at the range of 10 nm. The antimicrobial activity of ZnO nanoparticles synthesized using the *Ch. majus* extract was tested against standard strains of bacteria (*Staphylococcus aureus* NCTC 4163, *Pseudomonas aeruginosa* NCTC 6749, *Escherichia coli* ATCC 25922)*,* yeast (*Candida albicans* ATCC 10231), filamentous fungi (molds: *Aspergillus niger* ATCC 16404, dermatophytes: *Trichophyton rubrum* ATCC 28188), clinical strains of bacteria (*Escherichia coli*, *Pseudomonas aeruginosa, Staphylococcus aureus*) and yeast (*Candida albicans*). The study showed that zinc oxide nanoparticles were excellent antimicrobial agents. What is more, biologically synthesized ZnO nanoparticles demonstrate high efficiency in treatment of human non-small cell lung cancer A549.

## Introduction

Nanotechnology belongs to the fastest growing technologies in the world (Gao et al. 2013, Rico and White 2014). Nanosized materials have been an important subject both in basic and applied science (Jayaseelan et al. 2012). Nanoparticles are widely applied in medicine, the environment, and industry. Among nanoparticles, a highly significant and promising inorganic material is Zinc oxide (ZnO). Zinc oxide nanoparticles belong to the group of multifunctional inorganic nanoparticles. These metal nanoparticles have found a wide use in numerous industrial areas, such as solar cells, UV light-emitting devices, gas sensors, photocatalysts, pharmaceutical and cosmetic industries (Sangeetha et al. 2011). Zinc oxide nanoparticles (ZnO NPs) have been the subject of a considerable interest due to their unique antibacterial, antifungal and photochemical properties. ZnO nanoparticles have also been studied for possible applications in medicine. They exhibit a high degree of cancer cell selectivity and they are able to surpass the therapeutic indices of some commonly used chemotherapeutic agents (Hanley et al. 2008). There are various procedures for preparing ZnO nanoparticles, such as the chemical vapor, solvothermal, high temperature, direct precipitation, sol-gel and hydrothermal method (Suresh et al. 2015).

Chemical methods for synthesizing nanoparticles result in the presence of some toxic chemicals adsorbed on the surface, which may have a negative impact in medical application. Due to the growing awareness of green chemistry and other biological processes, there has been developed an eco-friendly approach to the synthesis of nanoparticles (Sangeetha et al. 2011). In order to ensure eco-friendly production of nanoparticles, biological organisms – such as microorganisms, plant extract or plant biomass – could be used instead of chemical and physical methods (Bhattacharya and Rajinder 2005). Green synthesis of nanoparticles using natural means like plants or algae (phytosynthesis) is also extensively discussed (Mohanpuria et al. 2008). As a result, environmentally friendly synthetic approaches have recently attracted much attention. In the literature, there can be found examples of the synthesis of zinc oxide nanoparticles using plants, such as *Plectranthus amboinicus* (Vijayakumar et al. 2015), *Cassia fistula* (Suresh et al. 2015), *Passiflora foetida* (Shekhawat et al. 2014), *Hibiscus rosa-sinensis* (Sharmila Devi and Gayathri 2014), *Ocinum basilicum* (Salam et al. 2014), *Cassia auriculata* (Koluru and Sharada 2014), *Vitex negundo* (Ambika and Sundrarajan 2015), *Aspalathus linearis* (Diallo et al. 2015) or *Solanum nigrum* (Ramesh et al. 2015).

In this work, the synthesis of ZnO nanoparticles was performed using the extract derived from *Chelidonium majus. Ch. majus* is naturally found in Europe, Asia and South America. As in the case of other representatives of the *Papaveraceae* family, the biological activity of the extracts from *Ch. majus* is determined mainly by alkaloids. They are chiefly isoquinoline alkaloids. Moreover, the plant contains sparteine, which is a quinolizidine alkaloid (a compound characteristic of the *Fabaceae* family) (Barton et al. 1999, Colombo and Bosisio 1996, Kopytko et al. 2005). The dominat alkaloids are chelidonine, chelerythrine, sanguinarine, beberine, coptisine and stylopine (Colombo and Bosisio 1996). Due to the characteristics of the biologically active compounds, the *Ch. majus* extract was used in the synthesis of ZnO nanoparticles. Besides, we tested the cytotoxicity and antibacterial activity of the synthesized ZnO nanoparticles.

## Materials and methods

### Synthesis of ZnO nanoparticles

Fresh and healthy samples of *Ch. majus* were collected in Wielkopolska region (Poland). *Ch. majus* was washed two times in distilled water. Then, it was air-dried for 30 days at room temperature. To 1.5 g powdered of *Ch. maju*s, there were added 100 ml of double distilled water. The solution was boiled and stirred for 1 h at the temperature of 90 °C. Subsequently, 50 ml of water extract were added to 5 g of Zn(NO_3_)_2_. The solution was stirred for 4 h, at the temperature of 75 °C. After that time, the UV-absorption spectrum of the synthesized ZnO nanoparticles was monitored. The obtained precipitate was dried in a hot air oven at 90 °C for 8 h.

### Characterization techniques

The maximum absorbance of the sample was measured with the use of UV-Visible spectrophotometry. The analysis of optical property of ZnO nanoparticles was made using ultraviolet and visible absorption spectroscopy (spectrophotometer Cary E 500) in the range of 250 nm–600 nm. The binding properties of ZnO nanoparticles were determined using FTIR analysis. In order to characterize the ZnO nanoparticles, there was conducted Fourier transform infrared spectroscopy (FTIR) analysis of the dried powder of the synthesized ZnO nanoparticles using Perkin Elmer Spectrum 1000, in attenuated total reflection mode and using spectral range of 4000–380 cm − 1, with a resolution of 4 cm − 1.

The shape, size and microstructures of the synthesized nanoparticles were determined with the use of a Transmission Electron Microscope JEOL JEM 1200 EXII, operating at 80 kV. The study was carried out in the tapping mode, using the atomic force microscope INTEGRA SPECTRA SOLAR of NT-MDT brand and measurement tips dedicated for NSGO1 high-resolution measurements. The resonance frequency of the tips ranged from 87 to 230 kHz. The force constant ranged from 1.45 to 15.1 N/m, and the scanning area was 10 μm × 10 μm. Within the scanning area, there were 1000 × 1000 scanning points. Before the measurement, the sample was placed on silicone substrate. It remained on the substrate until the solvent evaporated. X-ray diffraction studies of the ZnO nanoparticles were carried out using a BRUKER D8 ADVANCE brand *-2* configuration (generator-detector) x-ray tube copper S = 1.54 A and LYNXEYE PDS detector. The pictures of ZnO nanoparticles were prepared by means of scanning electron microscopy (SU3500), Hitachi with spectral imaging system Thermo Scientific NSS (EDS), detector tape (BSE-3D), acceleration voltage (15.0 kV), working distance (11.6 mm), pressure (in the case of variable vacuum conditions)(40 Pa).

### Antimicrobial activity of synthesized ZnO nanoparticles

Antimicrobial activity of biosynthesized ZnO nanoparticles using the *Ch. majus* extract was tested against standard strains of bacteria (*Staphylococcus aureus* NCTC 4163, *Pseudomonas aeruginosa* NCTC 6749, *Escherichia coli* ATCC 25922)*,* yeast (*Candida albicans* ATCC 10231), filamentous fungi (molds: *Aspergillus niger* ATCC 16404, dermatophytes: *Trichophyton rubrum* ATCC 28188), clinical strains of bacteria (*Escherichia coli*, *Pseudomonas aeruginosa, Staphylococcus aureus*) and yeast (*Candida albicans*). Bacterial and *C. albicans* strains were stored in Microbank *cryogenic vials* (ProLabDiagnostics, Canada) at −70 °C ± 10 °C. The filamentous fungi were maintained on Sabouraud dextrose agar (SDA; Merck, Germany) at 10 °C. The bacterial and *C. albicans* cultures were grown in Brain Heart Infusion broth (BHI; bioMerieux, France) at 34 °C for 18 h. Filamentous fungi were inoculated on Sabouraud dextrose agar and incubated at 34 °C for 5 days to 3 weeks for adequate sporulation. After incubation each culture were diluted in suitable liquid medium (bacteria - Mueller–Hinton broth, MHB, Oxoid, UK; fungi - Sabouraud dextrose broth, SDB, Merck, Germany) to obtain a final suspension containing about 1 × 10^6^ CFU/ml – bacteria and *C. albicans* and 2 × 10^6^ CFU/ml – filamentous fungi.

The antimicrobial activity of ZnO nanoparticles synthesized using the *Ch. majus* extract was assessed by determining the minimal inhibitory concentration (MIC), minimal bactericidal concentration (MBC) and minimal fungicidal concentrations (MFC). The MIC of products was studied by employing a macrodilution method, using: Mueller–Hinton broth (MHB) - bacteria and Sabouraud dextrose broth (SDB) - fungi.

The solution of biosynthesized ZnO nanoparticles was two-fold serially diluted in a culture broth to concentrations ranging from 5187 μM to 10 μM. In the study, 1 ml of each dilution and sterile nutrient broth (for growth control) was dispensed into tubes. Each tube and tube containing only nutrient broth (growth control) was inoculated with 1 ml of a microbial inoculum. All tested tubes were incubated at 34 ± 1 °C for 18 h – bacteria and *C. albicans* or 72 h – filamentous fungi. The tubes were than examined for evidence of growth and MICs values were determined as the lowest concentration at which visible growth was inhibited. MBC and MFC concentration was determined as an extension of the MIC test. For this, every tubes that demonstrated no growth (concentration equal to and greater than the MIC) were subcultured onto an agar medium: Typcase soy agar (TSA; BioMerieux) – bacteria; SDA - fungi. The plates were incubated at 34 ± 1 °C for 18 h – bacteria and *C. albicans* or 72 h – filamentous fungi*.* The MBC/MFC was defined as the lowest concentration at which no growth was observed. Control experiments were carried out under similar condition by using extract of *Ch. majus.* Amikacin (for bacteria) and nystatin (for fungi) were used as standard substances.

### Evaluation of cell proliferative activity of synthesized ZnO nanoparticles

#### Established cell lines

Much studies has shown that ZnO NPs cause cytotoxicity to many types of cells, such as osteoblast cancer cells, human bronchial epithelial cells (BEAS-2B), human kidney cells, human alveolar adenocarcinoma cells, human hepatocytes, and embryonic kidney cells (Kang et al. 2013). In this study, the influence of green synthesized ZnO nanoparticles using the *Ch. majus* extract on human cells *in vitro* was evaluated with use two established human cell lines. For this purpose the adherent fibroblast cells CCD-39Lu (ATCC® CRL-1498™) isolated from lungs and adherent epithelial cells of human non-small cell lung cancer A549 (ATCC ® CCL-185™) were used. Cell lines were grown on 24-Well flat-bottom plates (TC-PLATE 24 well, Greiner) in RPMI-1640 medium supplemented with 10% FBS, 2 mM L-glutamine without antibiotics. Cells were allowed at 37 °C in an incubator in a humidified atmosphere of 5% CO_2_.

#### Assessment of the cell cycle with use propidium iodide (PI)

Cells were cultured in the presence of a test substance for 24, 48 and 72 h. Proliferative activity was tested on the basis of the percentage of cells in S phase of the cell cycle. Additionally, the percentage of cells in the of green synthesized ZnO nanoparticles using the *Ch. majus* extract/M phase and percentage of dead cells was assessed. Percentage of cells was determined on the basis of the mean fluorescence intensity (MFI) emitted by fluorochrome named propidium iodide (PI), which intercalate into DNA of replicating cells. Intensity of the fluorescence emitted by PI is proportional to the proliferative activity.

Cell suspensions were transferred on to culture plates in concentration 4 × 10^4^ cells per well. After 24 h, when the cells adhered to the surface of the plate, the culture medium was changed and the tested substance in suspension with fresh medium were added in the following quantities: 100 μM, 10 μM and 1 μM. The control solution was a fully supplemented culture medium without test substance. Cells were incubated in triplicates for 24, 48, and 72 h. For the test with PI, cells were harvested from plates and resuspended in 600 μl of cold permeabilization buffer containing: RPMI 1640 culture medium, 2% fetal bovine serum (FBS) and 1% saponin (Sigma). After 30 min incubation, cells were centrifuged at 400 g for 5 min at 4 °C. Next, cell pellets were resuspended in 1 ml cold PBS containing 10 mg/ml propidiumiodide (Sigma) and 100 U/ml Rnase enzyme (Boehringer Mannheim) and incubated for 30 min at 4 °C protected from light. Finally, the samples were added to acquisition with use of flow cytometer FACS Canto (BectonDickinson), and percentages of cells in each phases of cell cycle were calculated using FACSDiva software (BectonDickinson).

##### Statistical analysis

Results received in the course of study were analyzed statistically. For this purpose were calculated nonparametric tests of Kruskal-Wallis and Friedman with Kendall coefficient of concordance.

## Results and discussion

### Characterization of ZnO nanoparticles

**Fig. 1:**
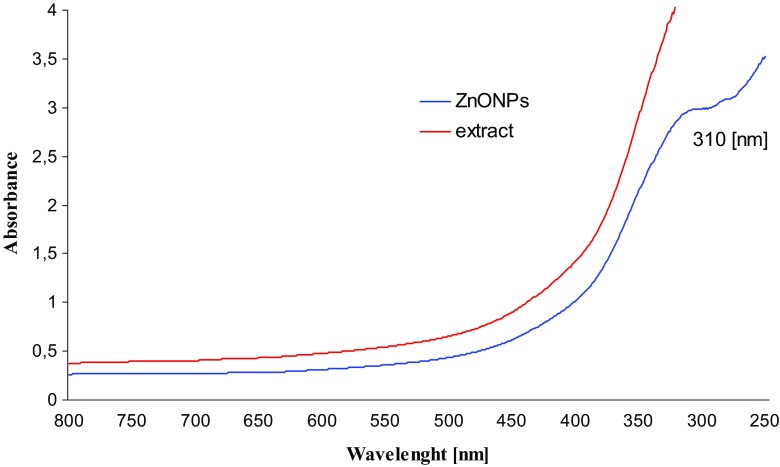
UV–visible spectra of biosynthesized ZnO nanoparticles and *Ch. majus* extract

The formation and stability of ZnO nanoparticles were followed by UV–Vis spectrophotometry. The UV-absorption spectrum of the synthesized ZnO nanoparticles was monitored at 4 h after preparation. Figure 1 shows UV–visible spectra of ZnO nanoparticles (ZnONPs) and *Ch. majus* extract. The presence of ZnO nanoparticles synthesized biologically with the use of the extract from *Ch. majus* was confirmed by the maximum absorption of about 310 nm, which is a characteristic band of pure ZnO (Wahab et al. 2014). Moreover, no other peak was observed in the spectrum, which again confirms that the synthesized products are pure ZnO (Wahab et al. 2009; Wahab et al. 2007). The research conducted by Pandurangan et al. (2016) showed a wide absorption of ZnO-NPs below 400 nm. Saraswathi et al. (2017) presented the absorption spectrum of ZnO-NPs with strong absorption band at about 373 nm. Ali et al. (2016) have reported the absorption peak of ZnO NP at 375 nm. According to Elumalai and Velmurugan (2015) the ZnO nanoparticles were characterized by a maximum absorbance peak at 370 nm.

**Fig. 2:**
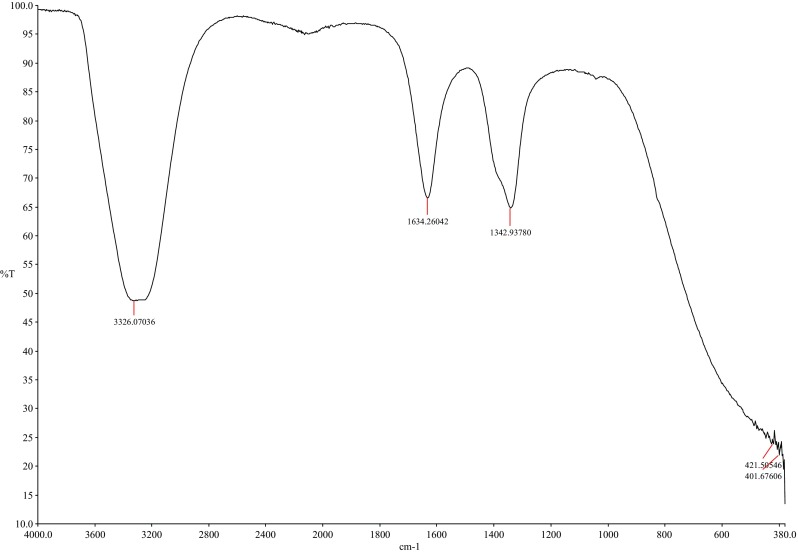
FTIR spectra of ZnO nanoparticles biosynthesized using of *Ch. majus* extract

FT-IR measurement was carried out in the wave number range from 380 to 4000 cm − 1 using the KBr method, at room temperature, as shown in Fig. 2. The strong peaks were observed at 3326 cm − 1, 1634 cm − 1, 1342 cm − 1, 421 cm − 1 and 401 cm − 1. The strong absorption peak at 3326 cm − 1 corresponds to -OH stretching and the aliphatic methylene group -C-H stretching. The most intense band at 1635 cm − 1 represents vibrations C = O, typical of the structure of flavonoids which can be found in the *Ch. majus* extract. The absorption band at 1342 cm-1 is related to CH bending vibrations of the aromatic tertiary amine group. The spectrum showed bands at 421 cm − 1 and 401 cm − 1, which indicated the formation of metal–oxygen stretching of ZnO nanostructure.

Fourier transform infrared spectroscopy (FTIR) studies confirmed the presence of bioactive compounds in *Ch. majus*, which served as reducing and capping agents for ZnO nanoparticles. According to Saraswathi et al. (2017) the region between 400 and 600 cm − 1 is attributed to Zn-O group. The similar results were presented by Krishna et al. (2016). The authors have reported that the transmittance band at 445 cm − 1 corresponded to the Zn–O bonding and confirmed the presence of ZnO particles. Murugan et al. (2017) demonstrated the high intensity band around 440 cm-1 due to stretching mode of zinc and oxygen bond.

**Fig. 3:**
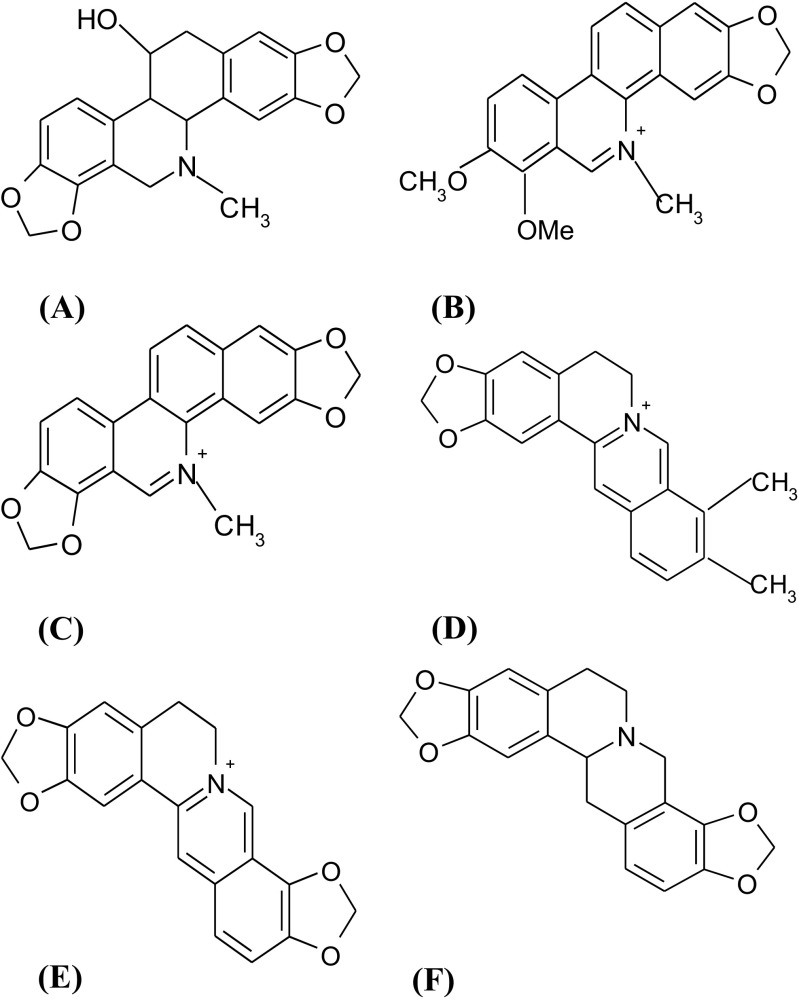
Chemical structure of main alkaloids contained in *Ch. majus*: **a** chelidonine, **b** chelerythrine, **c** sanguinarine, **d** berberine, **e** coptisine, **f** stylopine

Based on the literature, *Ch. majus* contains such compounds as sparteine, oxysanguinarine, chelirubine, chelidamine, methoxychelidonine, homochelidonine, chelidonic acid, ascorbic acid, malic acid, citric acid, succinic acid, carotene, essential oil, flavonoids, saponins, tannins, and mineral salts. Moreover, the reserve substances in the cells of *Ch. majus* do not have the form of starch but of glycogen (characteristic of animal tissues). *Ch. majus* latex is rich in many isoquinoline alkaloids (more than 20 have been identified), which belong to three main groups: (a) benzophenanthridines, like chelidonine, sanguinarine and chelerythrine, (b) protopine and derivatives, and (c) protoberberines, like berberine and coptisine (Carmo Barreto et al. 2003). From the chemical perspective, alkaloids are found mainly in the form of salts with citric, malic, succinic and chelidonic acid. The dominant alkaloids present in *Ch. majus* include: chelidonine, chelerythrine, sanguinarine, berberine, coptisine and stylopine. Chelidonine, chelerythrine and sanguinarine are quaternary benzo [c]phenanthridine alkaloids (QBAs) and display a wide spectrum of attractive biological activities. Figure 3 presents the chemical structure of main alkaloids contained in *Ch. majus* ***.*** They are regularly used in folk medicine as antimicrobial, antifungal, and anti-inflammatory agents.

### Morphological observations of ZnO nanoparticles

**Fig. 4:**
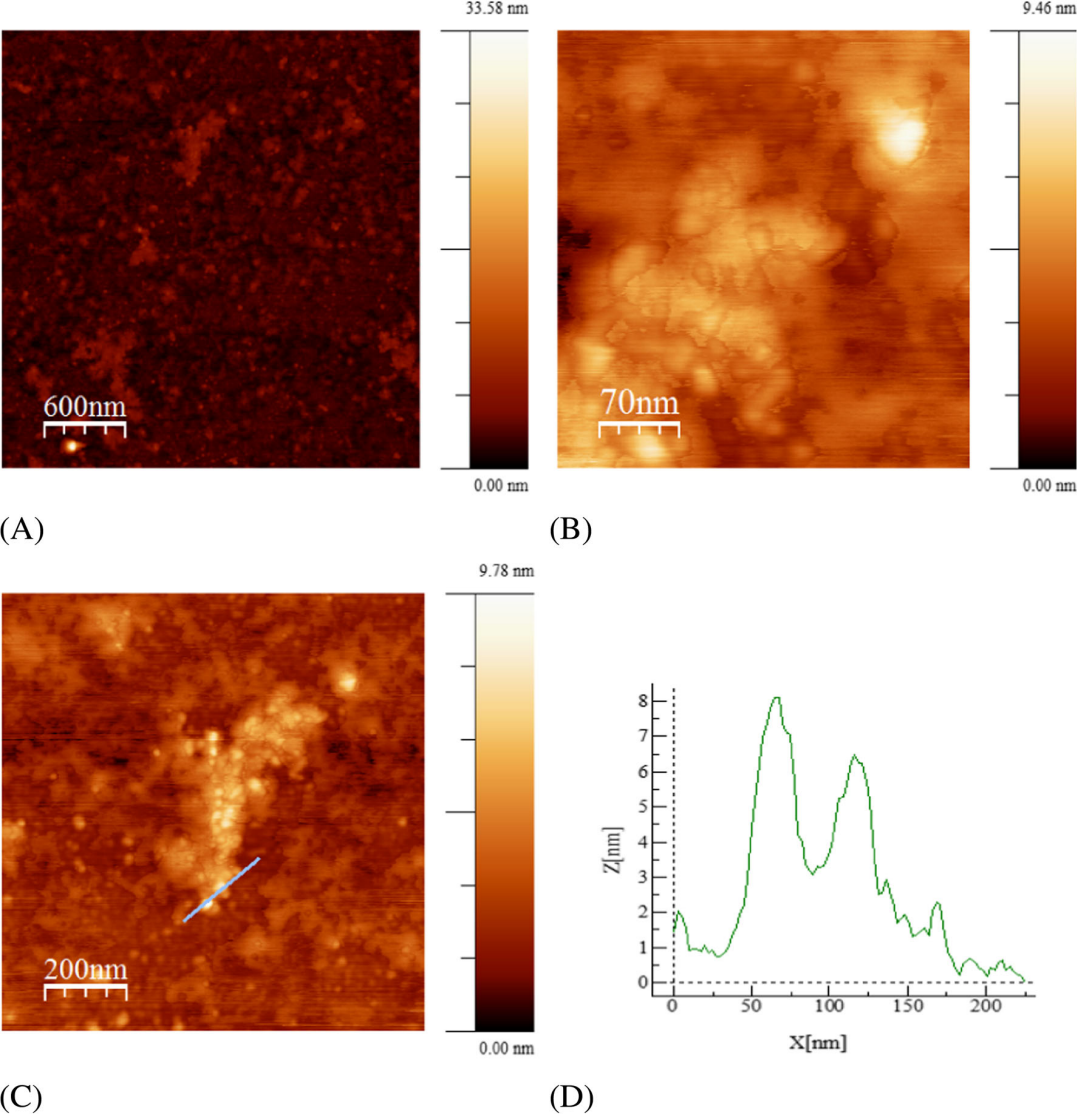
AFM image of ZnO nanoparticles biosynthesized using of *Ch. majus* extract (**a**) the topography 3 μm × 3 μm, (**b**) the topography 350 nm × 350 nm, (**c**) the topography 1 μm × 1 μm with the profile of nanoparticles, (**d**) the profile for the topography 1 μm × 1 μm

Atomic force microscopy (AFM) was used to observe the samples surface morphology and roughness. The obtained sample topographies were shown in Fig. 4. The diameters of a hundred random particles were measured on Z axis (height). The determined of the synthesized ZnO nanoparticle diameter was 8 nm.

**Fig. 5:**
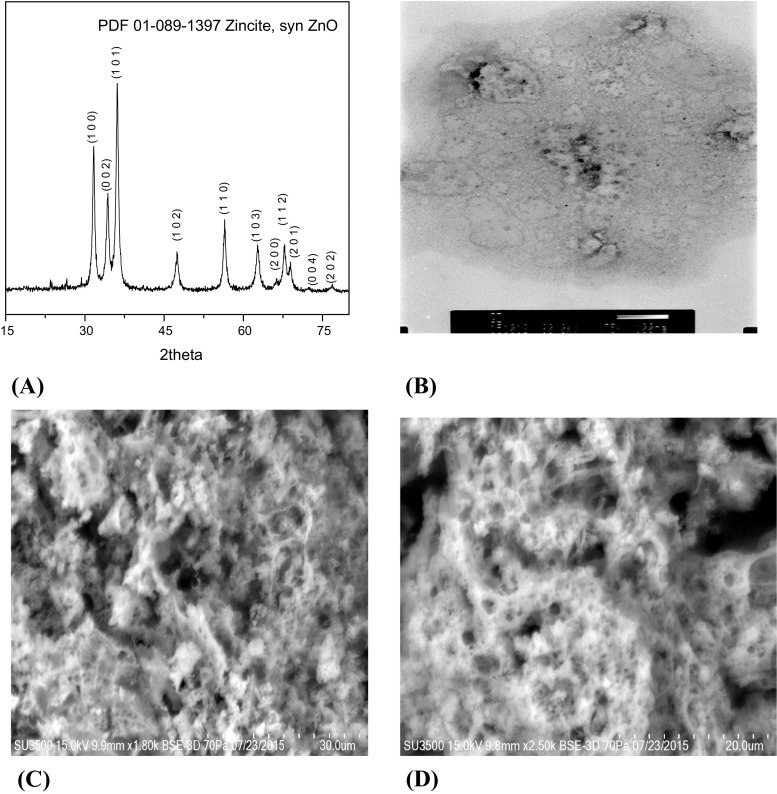
**a** XRD pattern, **b** TEM image and **c**, **d** SEM images of ZnO nanoparticles biosynthesized using of *Ch. majus* extract (the scale bar is 30 and 20 μm)

X-ray diffraction was taken to further confirm ZnO phase of the nanoparticles. Figure 5(a) shows the X-ray diffraction profile of ZnO nanoparticles synthesized using the *Ch. majus* extract. The XRD peaks were identified as (100), (002), (101), (102), (110), (103), (200), (112), (201), (004) and (202). The diffraction pattern agreed with the standard Joint Committee on Powder Diffraction Standards (JCPDS) No. 89–1397. The XRD spectrum suggested that ZnO nanoparticles were crystalline and purity. The size of synthesized ZnO nanoparticles using the *Ch. majus* extract was obtained by Debye-Scherrer’s formula:

$$ \mathrm{D}=\mathrm{K}\uplambda /\left(\upbeta \mathrm{cos}\uptheta \right) $$

where:

D: the crystal size,
λ: the wavelength of the X-ray radiation (λ = 0.15406 nm) for CuKα,
K: usually taken as 0.89,
β: the line width at half-maximum height (Vijayalakshmi and Rajendran 2012). The crystal sizes calculated using Scherrer’s formula were about 10 nm.

Transmission Electron Microscope Analysis (TEM) measurements were used to determine the size, shape and morphological features of the synthesized ZnO nanoparticles using the *Ch. majus* extract. Figure 5(b) shows the transmission electron micrograph of the biosynthesized ZnO nanoparticles. Moreover, Fig. 5(b) (magnification 100,000 x) confirmed that the size of the synthesized ZnO nanoparticles was less than 10 nm. Moreover, the transmission electron microscopy image presents the spherical structure of ZnO nanoparticles. TEM image confirmed the results obtained from Atomic Force Microscopy (AFM) studies.

The morphology of synthesized ZnO NPs was observed by scanning electron microscopy (SEM). SEM characterization carried out at different magnifications is shown in Fig. 5(c) i (d). Figures 5 c-d present the scanning electron microscopy (SEM) images of ZnO nanoparticles synthesized using the *Ch. majus* extract, where the scale bar is (c) 30 μm and (d) 20 μm. The size of synthesized ZnO nanoparticles is consistent with the measurement of particle diameter by means of XRD, TEM, AFM analysis.

**Fig. 6:**
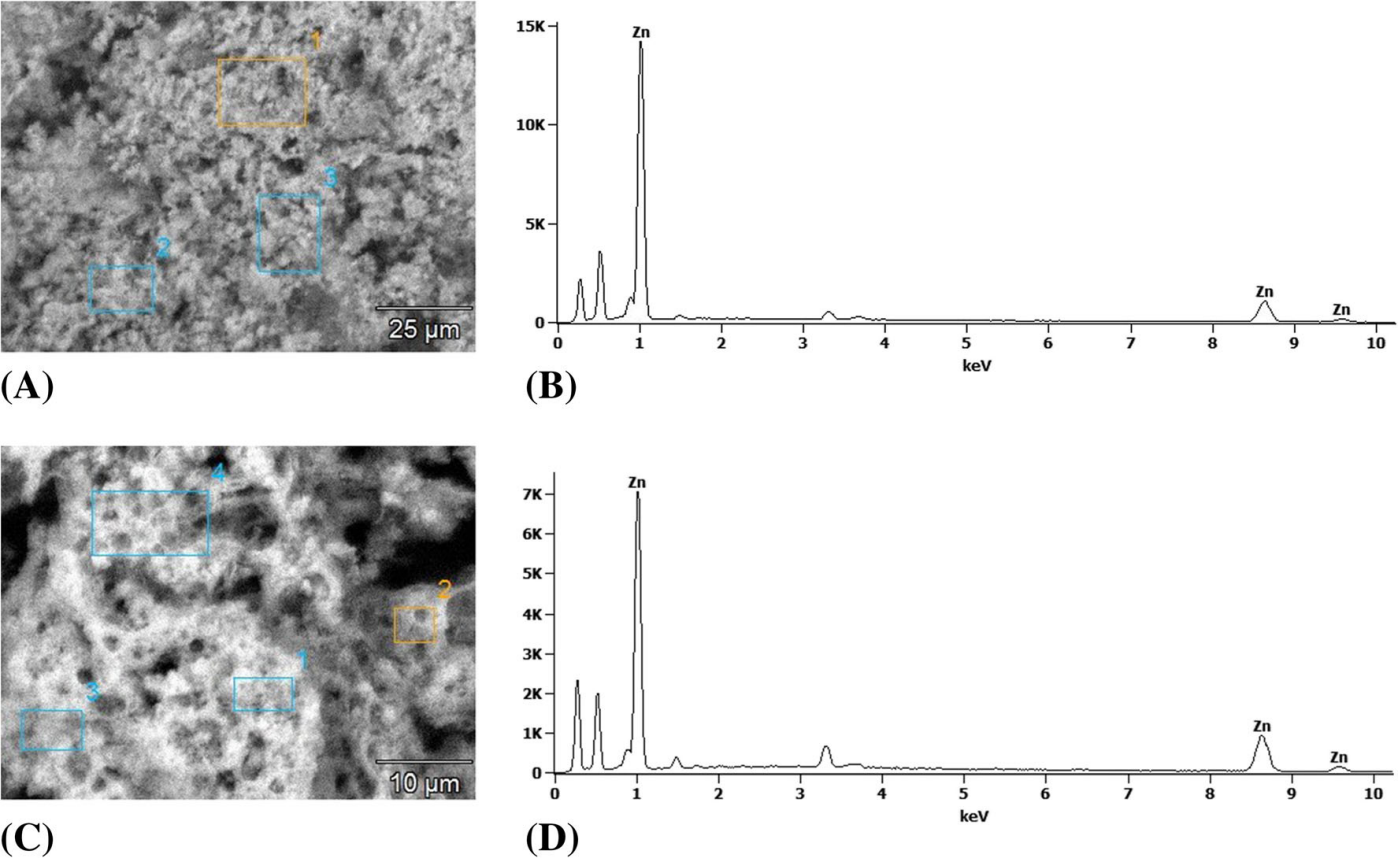
SEM images of the synthesized ZnO nanoparticles biosynthesized using of *Ch. majus* extract where (**a**) the scale bar is 25 μm and (**c**) the scale bar is 10 μm and EDS profiles (**b**) (**d**)

Figure 6 presents the SEM images of ZnO nanoparticles biosynthesized using the *Ch. majus* extract, where (a) the scale bar is 25 μm and (c) the scale bar is 10 μm. Moreover, Fig. 6 (b) and (d) present three peaks between 1 kV and 10 kV, which confirmed the presence of high purity zinc oxide nanoparticles in the analyzed sample.

### Antibacterial activity of ZnO nanoparticles

Antimicrobial activity of synthesized ZnO nanoparticles against both standard and clinical strains of pathogenic bacteria – *Staphylococcus aureus, Escherichia coli, Pseudomonas aeruginosa* and fungi – *Candida albicans, Trichophyton rubrum, Aspergillus niger* was investigated. Antibacterial activity results revealed that ZnO nanoparticles acted as excellent antibacterial agents against both Gram-positive and Gram-negative bacteria (Table 1). According to the obtained results standard strain of *S. aureus* exhibited the highest sensitivity toward ZnO nanoparticles, and the MIC and MBC value were comparable to those obtained for amikacin used as reference antibacterials. The ZnO NPs were slightly less active against clinical strains of *S. aureus* and Gram-negative rods of *E. coli.* The strains of *P. aeruginosa,* the bacterium that exhibit natural resistance to many antimicrobials, showed the lowest sensitivity to synthesized ZnO NPs but the MIC and MBC value were still low. The obtained values of MIC in the range of 80 μM to 120 μM demonstrate, that ZnO nanoparticles prepared by the described method have high antifungal activity and the obtained MIC values remain below or slightly above (*T. rubrum*) the MIC of nystatin. The fungicidal effect against standard and clinical strains of *C. albicans* and *A. niger* was not observed at all tested concentrations whereas MFC for *T. rubrum* was only 1.6 fold higher than MFC obtained for nystatin. Extract of *Ch. majus* did not show any inhibition in control study (data no shown) which suggested that the antimicrobial activity was specifically due to ZnO NPs.

**Table 1:** Antimicrobial activity of ZnO nanoparticles synthesized using of *Ch. majus* extract

Microorganisms	ZnO nanoparticles synthesized using of *Ch. majus* extract		Amikacin		Nystatin	
	MIC [μM]	MBC/MFC [μM]	MIC [μM]	MBC [μM]	MIC [μM]	MFC [μM]
*Staphylococcus aureus* NCTC 4163	20	40	25	25	NT	NT
*Pseudomonas aeruginosa* NCTC 6749	120	640	25	25	NT	NT
*Escherichia coli* ATCC 25922	80	80	25	25	NT	NT
*Escherichia coli*	80	80	25	25	NT	NT
*Staphylococcus aureus*	40	120	25	25	NT	NT
*Pseudomonas aeruginosa*	120	640	50	50	NT	NT
*Candida albicans* ATCC 10231	80	> 2590	NT	NT	100	200
*Candida albicans*	120	> 2590	NT	NT	200	400
*Trichophyton rubrum* ATCC 28188	120	640	NT	NT	100	400
*Aspergillus niger* ATCC 16404	80	>2590	NT	NT	400	12,640

*MIC-* Minimal Inhibitory Concentration,

*MBC-* Minimal Bactericidal Concentration,

*MFC-* Minimal Fungicidal Concentration,

*NT-*not tested

The current investigation showed that ZnO nanoparticles have a broad spectrum of antimicrobial activity. This research provides valuable preliminary efficacy data of ZnO NPs for control of microbial growth. The antimicrobial activity of the nanoparticles is known to be a function of interaction between nanoparticles higher surface area and microorganisms, i.e., large surface area of the nanoparticles enhances microbes to carry out a broad range of probable antimicrobial activities chyba powinno być: large surface area of the nanoparticles enhances a probability of broad range of antimicrobial activities (Martínez-Gutierrez et al. 2010). According to the literature, the antibacterial activity of ZnO is caused by the production of active oxygen species (Janaki et al. 2015) including hydrogen peroxide (H_2_O_2_), a strong oxidizing agent (Xie et al. 2011). According to Feris et al. (2010) the toxic effects of ZnO nanoparticles on the pathogenic species of bacteria are increased due to the prolonged contact between the bacterium cell membrane and ZnO nanoparticles. According to the study by Xie et al. (2011), the mechanism of ZnO inactivation of bacteria involves direct interaction between ZnO nanoparticles and cell surfaces. It is well known that ZnO is a polar crystal, Zn^2+^ lies within a tetrahedral group of four oxygen ions. Zinc and oxygen atoms are arranged alternatively along the c-axis and the top surfaces are Zn terminated while the bottom surfaces are oxygen terminated (Shang et al. 2005). The majority of studies suggest that nanoparticles cause disruption of bacterial membranes probably by the production of reactive oxygen species (ROS) such as superoxide and hydroxyl radicals. As a particle approaches near the membrane, a potential called zeta potential is generated. This is different for various nanoparticles. ZnO nanoparticles have positive zeta potential at their surface (Brayner et al. 2006). In effect, oxidative stress in bacterial cells contributes to the inhibition of cell growth and eventually to cell death. Several studies have believed that the leaked Zn^2+^ into growth media responsible for ZnO nanotoxicity and the dissolution of ZnO-NPs into Zn^2+^ were found as size dependent (Saliani et al. 2016). Kasemets et al. (2009) have shown that the release of Zn^2+^ ions was a logical cause of ZnO toxicity toward *S. cerevisiae*. According to this hypothesis, ZnO-NPs toxicity is referred to the solubility of Zn^2+^ in the medium including the yeast.

The results presented in this study confirm the previous findings that zinc nanoparticles have significant inhibitory effect on bacterial growth. This is consistent with our previous report that zinc nanoparticles have significant inhibitory effect on bacterial growth. It should be noticed that Gram-negative bacterial strains of *E. coli* and especially *P. aeruginosa* were less sensitive to prepared ZnO NPs than Gram-positive bacterial strains of *S. aureus*. Our finding is in agreement with other investigators, who reported that the ZnO nanoparticles are more effective against Gram-positive bacterial strains than Gram-negative bacterial strains. It is well known that Gram-negative bacteria are more resistant to antimicrobials than Gram-positive due to the differences in composition of the cell wall and presence of an outer membrane in Gram-negative bacteria (Sirelkhatim et al. 2015). It has been reported that ZnO nanoparticles are more toxic for the prokaryotic cells than more sophisticated eukaryotic cells. Thus bacteria are killed with lower ZnO concentrations than fungi. The toxicity mechanism of antimicrobial activity of ZnO NPs is not completely known and still requiring deep explanations. Mechanisms that have been put forward in the literature are both the production of reactive oxygen species and the accumulation of nanoparticles in the cytoplasm or on the outer membranes. This multiple mechanisms may explain the excellent activity of ZnO NPs against bacteria. The higher resistance of fungi to ZnO compared to bacteria may come from not only the differences in the cell structure but also from the fact that fungi are less sensitive to ROS. The activity of ZnO on the fungi was fungistatic in the concentration range studied but fungicidal effect was observed only against *Trichophyton rubrum.*

Overall, our results suggest that ZnO nanoparticles could be developed as antimicrobial agents against a wide range of microorganisms to control and prevent the grown spreading of microbes. Additionally, the extract of *Ch. majus*, already used in the reaction, contains a large number of alkaloids and polyphenols; therefore, it is known for its antimicrobial activity (Meng et al. 2009; Nawrot et al. 2007; Zuo et al. 2011). Other antifungial compounds identified in the *Ch. majus* extract were the phenolic compounds rutoside, pcoumaric acid, ferulic acid, quercetol, and kaempherol. The mechanisms of action thought to be responsible for phenolic toxicity involve enzyme inhibition by the oxidized compounds, possibly through reaction with sulfhydryl groups or nonspecific interactions with the proteins (Arif et al. 2009).

### Evaluation of cell proliferative activity of f ZnO nanoparticles

**Fig. 7:**
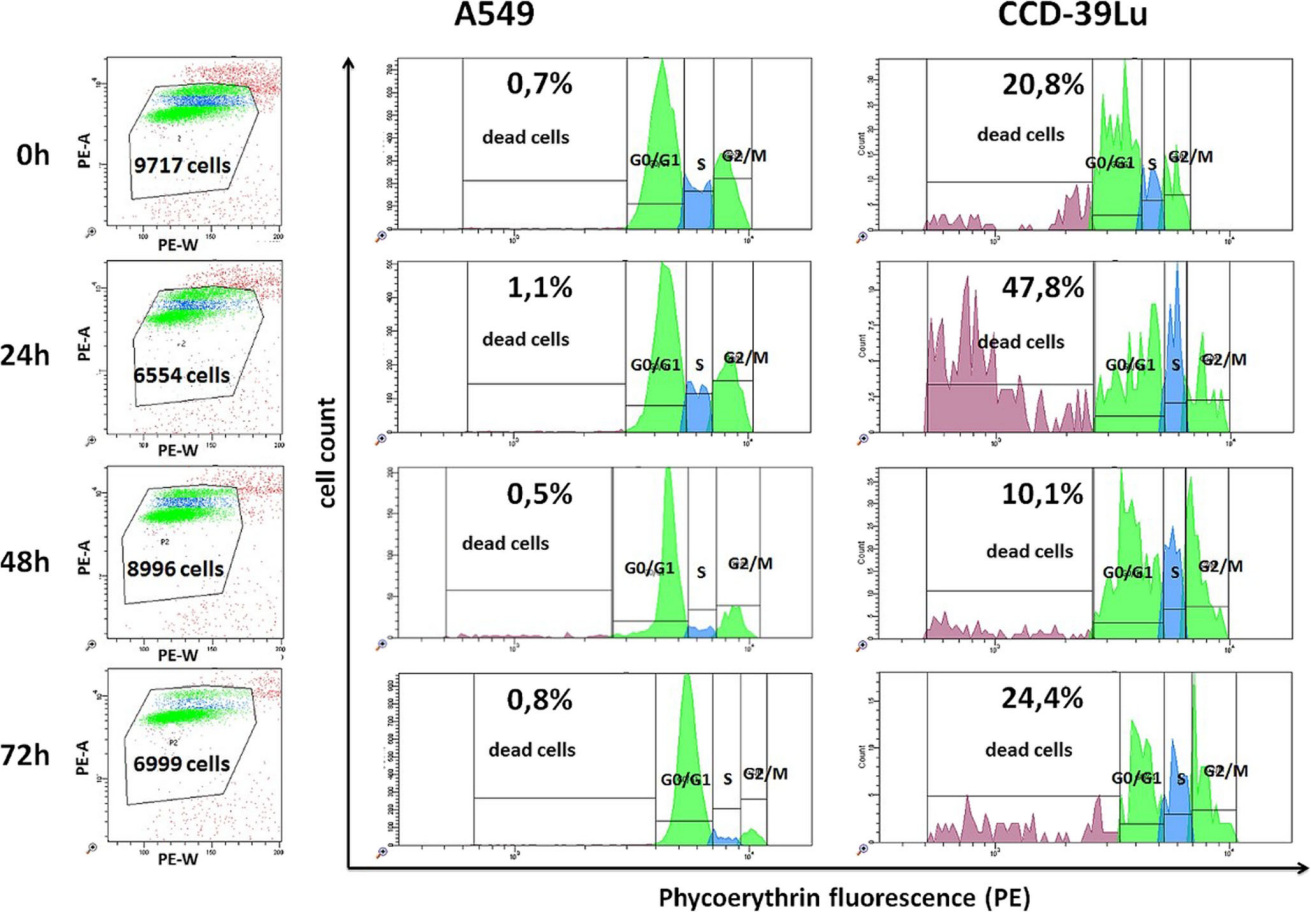
Scattergrams show the number of viable cells during different stages of culture in presence of 100 μM ZnO nanoparticles. Histograms represent the analysis of the cell cycle per unit of time. The marked regions present the respective phases of the cell cycle, emphasizing the percentage of dead cells

Obtained results indicate, that the biosynthesized ZnO nanoparticles may act on A549 and CCD-39Lu cells in different ways. The observed interactions were depended on the time of culture and the concentration of the substance. The biosynthesized ZnO nanoparticles using the *Ch. majus* extract were added to the cells simultaneously with culture medium in concentrations 1 μM, 10 μM and 100 μM, and cells were cultured up to 72 h. Detailed analysis performed after 24, 48 and 72 h of culture indicated different pattern of stimulation of A549 and CCD-39Lu cells. The assessment of the cell cycle in the S-phase revealed a different behavior of malignant A549 and non-malignant CCD-39Lu cells.In S-phase the cells replicate the DNA. Lung cancer cells after 24 and 48 h of culture more intensively proliferated than unstimulated control cells. However, after 72 h of culture the percentage of cells stimulated with the biosynthesized ZnO nanoparticles was comparable with level showed by control cells. CCD-39Lu fibroblasts, both in the 24 and 72 h of culture, indicated diminished proliferation activity as compare to the control cells. Only in the 48 h the control cells and cells stimulated with ZnO nanoparticles showed comparable level (Fig. 7).

**Fig. 8:**
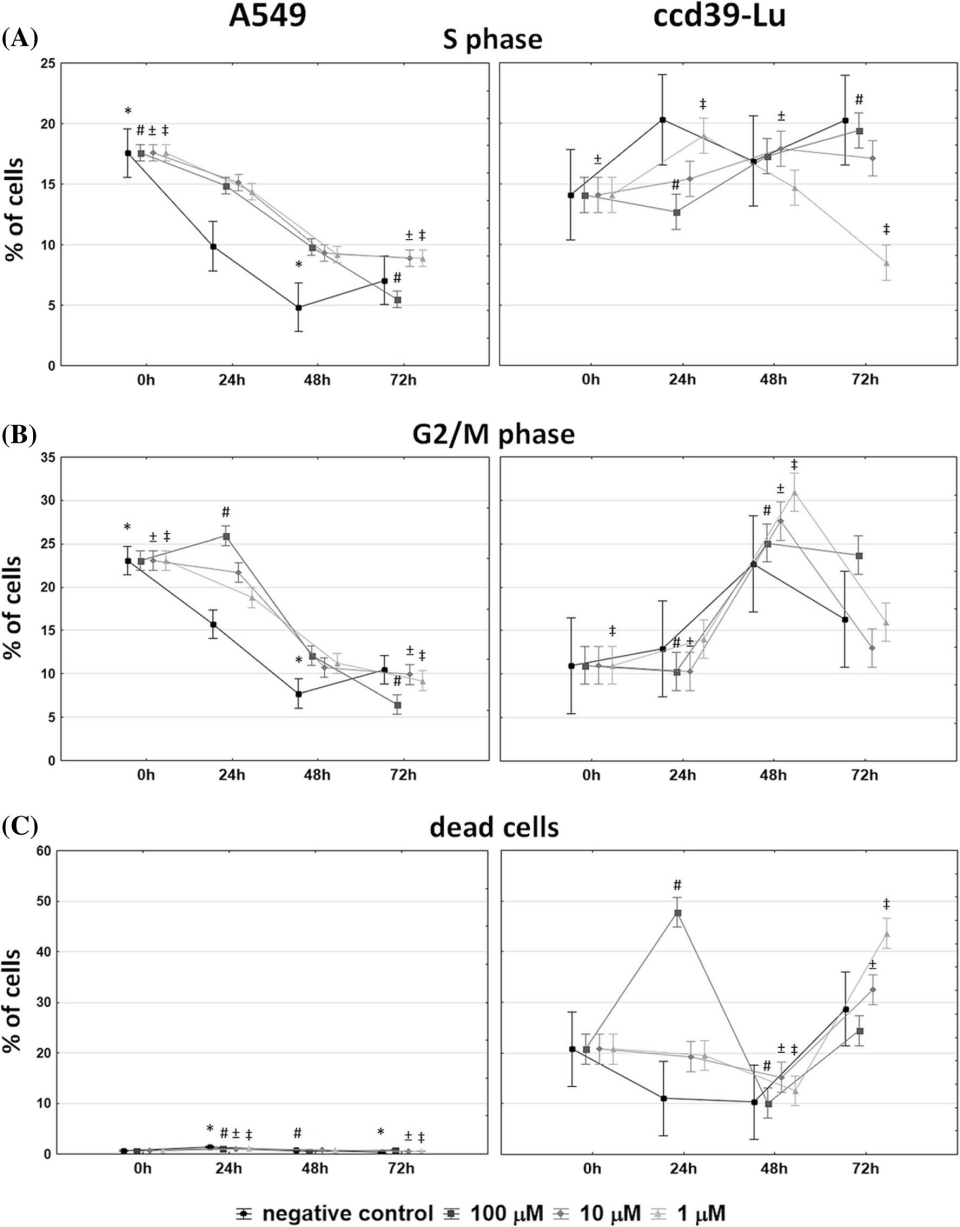
Percentage of A549 and CCD39-Lu cells cultured in the presence of ZnO nanoparticles in the S and G2/M phases of the cell cycle, and dead cells, measured in different periods of time(plots –means; error-bars – SD). Values statistically different at *p* < 0.05(* - negative control; # - 100μM; ± − 10μM; ‡ − 1μM) in Kruskal-Wallis test

The analysis of the cell cycle also was focused on the G2/M phase, and indicated a proportion of dead cells. Generally, in the G2/M phase percentage of A549 cells during the time of culture was decreased, while CCD-39Lu cells was increased. More particularly, in the G2/M phase the percentage of A549 cells in the 24 h of culture was directly proportional to the concentration of ZnO nanoparticles. When the concentration of the biosynthesized ZnO nanoparticles using the *Ch. majus* extract was higher, the higher percentage of cells was observed. In contrast, CCD-39Lucells in 48 h of culture showed inversely proportional relationship. The higher percentage of cells in the G2/M phase was observed when the concentration of the ZnO nanparticles was lower (Fig. 8).Vital functions of A549 cells under the influence of ZnO nanoparticles were not inhibited. The percentage of dead cells was not crossing the 2%. Generally, during performed evaluation the A549 cells showed lower number of dead cells as compared to the CCD-39Lu cells. However, in the 24 h of culture the CCD-39Lu cells under the influence of ZnO nanoparticles in 100 μM concentration exhibited maximum mortality, the value of which reached 50% (Fig. 8).

Results received in the course of study were analyzed statistically. For this purpose were calculated nonparametric tests of Kruskal-Wallis and Friedman with Kendall coefficient of concordance. Moreover, a Wilk’s multivariate analysis of variance was conducted. Results of Wilk’s tests for all studied values showed that the multivariate effect between applied ZnO concentrations was statistically significant. Using Kruskal–Wallis test by ranks was showed that differences in the percentages of cells at different hours of culture were statistically significant in a range of groups (Fig. 8).

Our results presented that the ZnO nanoparticles included in the *Ch. majus* extract possessed the restricted cytotoxic activity against non-small cell lung cancer. However, in the G2/M phase, the percentage of A549 cells at the 24th hour of the culture was directly proportional to the concentration of ZnO nanoparticles. Moreover, highest percentages of dead cells were observed at the highest concentration of ZnO. The observed initial increase of proliferation activity of the A549 cells was gradually silenced, whereas the percentage of dead cells was steadily increased. At last, the percentage of A549 cells in S phase of the cell cycle in the final of time reached to a value close to that observed in the control culture. The significantly stronger cytotoxic interaction of ZnO nanoparticles on CCD39Lu fibroblasts corresponds to the findings of other authors. Some authors suggested cytotoxicity of ZnO nanoparticles in several types of cancers. Guo et al. (2008) studied the synergistic cytotoxic effect of differently sized ZnO nanoparticles with the accompanying anticancer drug of daunorubicin on leukemia cell lines, and found it efficient to exert the synergistic cytotoxicity suppression on both leukemia cell lines under UV irradiation. The research conducted by Wahab et al. (2014) demonstrated that ZnO-NPs induced cytotoxicity and apoptosis in HepG2 and MCF-7 cancer cells. Pandurangan et al. (2016) showed that a ZnO nanoparticle had a cytotoxic effect on human cervical carcinoma cells. Mariappan et al. (2011) have reported that ZnO nanoparticles inhibit human myeloblastic leukemia cells and are less toxic to normal peripheral blood mononuclear cells. Wang et al. (2015) indicated that ZnO NPs have significant dose-dependent cytotoxic effect on human pulmonary adenocarcinoma cells LTEP-a-2. The study of Krishna et al. (2016) demonstrated the high anticancer activity of ZnO nanoparticles against prostate and breast cancer cell lines. Krishna et al. (2017) showed the anticancer activity of ZnO nanoparticles performed on two human cancer cell lines DU-145 (human prostate cell line) and Calu-6 (human pulmonary adenocarcinoma). Moreover, blood hemolysis studies conducted proved the bio compatibility of ZnO nanoparticles at varied concentrations.

The additional cytotoxic effect probably stemmed from the presence of alkaloid compounds in *Chelidonium majus*. *Ch. majus* alkaloids have been thoroughly studied and their potential application as anticancer agents has already been reported (Barnes et al. 2007; Colombo and Bosisio 1996; Kemeny-Beke et al. 2006). The quaternary benzo[c]phenanthridine alkaloids, sanguinarine and chelerythrine, from the *Ch. majus* extract, were good candidates for chemotherapeutic regimens of some carcinomas (Malikova et al. 2006). As indicated in previous researches, mitochondrion was the major cellular target of isoquinoline alkaloids in the induction of apoptosis, Kemeny et al. 2006, Slaninova et al. 2007). The mechanism of sanguinarine and chelerythrine towards mitochondria may be caused by the induction of reactive oxygen species (ROS) production, which leads to impairment of the integrity of plasma membrane and a quick development of necrotic processes (Chang et al. 2007; Kaminskyy et al. 2008).

## Conclusion

In this work, the extract of *Chelidonium majus* was used for the biological synthesis of ZnO nanoparticles. The techniques for measuring ZnO nanoparticles used in the study, such as UV–visible, Transmission Electron Microscopy (TEM), Atomic Force Microscopy (AFM) measurements, X-ray diffraction (XRD) and Scanning Electron Microscopy (SEM) with EDS analyzer, confirmed the presence of nanoparticles with the size of 10 nm. The synthesized ZnO nanoparticles presented good biocidal effect on all tested microorganisms. Also, the biosynthesized nanoparticles are an effective bioactive factor for inhibiting growth of both bacteria and fungi. What is more, the study offer opportunities for the search of an effective anti-cancer therapy against lung cancer cells.
